# Evaluation of a peer support group programme for vulnerable host population and refugees living with diabetes and/or hypertension in Lebanon: a before-after study

**DOI:** 10.1186/s13031-025-00646-4

**Published:** 2025-01-29

**Authors:** Leah Anku Sanga, Carla Njeim, Éimhín Ansbro, Rima Kighsro Naimi, Ali Ibrahim, Benjamin Schmid, Jasmin Lilian Diab, Jytte Roswall, Tim Clayton, Lars Bruun Larsen, Pablo Perel

**Affiliations:** 1 London School of Hygiene and Tropical Medicine, Department of Non-Communicable Diseases Epidemiology, Keppel street, London, WC1E 7HT UK; 2 Lebanese Red Cross, Beirut, Lebanon; 3 Danish Red Cross, Copenhagen, Denmark; 4 Institute for Migration Studies, School of Arts and Sciences, Lebanese American University, Beirut, Lebanon; 5 London School of Hygiene and Tropical Medicine, Department of Medical Statistics, London, UK

## Abstract

**Background:**

Non-communicable diseases (NCDs) are the leading cause of death globally, and many humanitarian crises occur in countries with high NCD burdens. Peer support is a promising approach to improve NCD care in these settings. However, evidence on peer support for people living with NCDs in humanitarian settings is limited. We evaluated the implementation of peer support groups (PSGs) for people with diabetes and/or hypertension as part of an integrated NCD care model in four primary care centers in Lebanon.

**Methods:**

Our objectives were to: (1) evaluate the reach of the PSGs; (2) evaluate the association of PSGs with patient-reported outcomes; and (3) evaluate the association of PSGs with clinical outcomes (blood pressure, HbA1c, and BMI). We used a before-after study design and included a control group for clinical outcomes. The PSG intervention began in December 2022 and was carried out in two waves. The first wave was implemented from December 2022 to July 2023, and the second wave from July 2023 to January 2024. For the control group on clinical outcomes, we used data collected from January 2023 to January 2024. We used routinely collected programmatic and administrative data. The patient reported outcomes (PROMs) were collected at baseline and at six months by trained volunteers for all PSG participants. We performed a before-after analysis of PROMs for all patients who completed the PSG sessions. T-tests were used to analyze the differences in PROMs from baseline. Change in PROMs, together with 95% confidence intervals (CIs), and p-values for the changes were reported. To assess the association between the implementation of the PSG strategy and changes in clinical outcomes, including systolic blood pressure (SBP), glycated hemoglobin A1c (HbA1c), and body mass index (BMI), analysis of covariance (ANCOVA) models were used, adjusting for age, sex, and the baseline values of the outcome being analyzed (baseline SBP and baseline HbA1c, respectively).

**Results:**

A total of 445 patients were approached for enrolment in wave 1, 259 (58%) consented, of whom 81 were enrolled. In wave 2, 169 patients were approached, 92 (54%) consented of whom 91 were enrolled. We found some statistical evidence that PSG improved certain PROMs, including potentially clinical meaningful improvements in overall quality of life (wave 1), physical quality of life (wave 1), social quality of life (wave 2), environmental quality of life (wave 1), adherence (wave 2), patient centeredness (wave 1), and exercise (wave 1). However, we did not find strong statistical evidence of an improvement in clinical outcomes (SBP, HbA1c, or BMI) in participants of the PSGs compared to the control group. We found differences in the association of PSGs and outcomes between the two waves.

**Conclusion:**

Our study showed mixed results. In terms of reach, over 50% of those approached consented to participate. Regarding the impact on PROMs, we observed improvements in most outcomes; however we found some statistical evidence only for some. We did not find strong statistical evidence of improvement in clinical outcomes compared to the control group. Differences between the two waves may be due to differences in the populations, the way the intervention was delivered, or the individuals implementing it. Additionally, as multiple outcomes were measured, some observed differences may be due to chance. We demonstrated that it is feasible to implement PSGs in humanitarian settings and found some statistical evidence of improvement in quality of life. Further studies should assess the implementation and impact of PSGs in ways that are well accepted by local stakeholders (including humanitarian actors and people living with NCDs) and are potentially amenable to scale-up.

**Supplementary Information:**

The online version contains supplementary material available at 10.1186/s13031-025-00646-4.

## Background

Non-communicable diseases (NCDs) are the leading cause of death globally, accounting for 75% of total deaths, most of which occur in low- and middle-income countries (LMICs) [1]. Most humanitarian crises also occur in LMICs, and many recent prolonged crises are taking place in middle income countries with high NCD burdens. Refugees and internally displaced persons living with NCDs are vulnerable to exacerbations due to the stresses of flight and displacement and they often face major barriers in accessing care [2].

The provision of NCD care in these settings is complicated by insecurity; limited health system resources, capacity and financing to deliver NCD care; as well as interrupted supply chains and population movement [3–5]. Humanitarian actors have gained increasing experience in managing NCDs over the last decade, developing guidance, tools and care models, largely focussing on getting the basic health system building blocks right. Standardisation, continuity of care and integration with national health systems and with other conditions, remain areas for improvement [6, 7]. Self-care, empowerment of people living with NCDs (PLWNCDs) and their families, and community-based models of care are key areas for development within models of NCD care in crises [6]. Existing guidelines (e.g. WHO package of essential non-communicable (PEN) disease interventions for primary health care and World Health Organisation (WHO) package for cardiovascular disease management in primary health care, WHO HEARTS) often focus on one-on-one consultations and on control of clinical outcomes. They tend to include little support or empowerment for patients to self-manage their NCD within their daily lives.

However, social relationships and self-care support have been shown to be critical for the well-being of PLWNCDs [8, 9]. Peer support has emerged as a promising community-based NCD care approach that utilises social relationships to promote self-care [10]. The term refers to support offered by a non-professional who shares lived experiences, such as a shared diagnosis or other relevant characteristics, with those they help [11]. A variety of potentially valuable peer support modalities have been used, which differ in their format (online, blended, in-person), choice of facilitator (peers, social workers, community member, volunteers), engagement with health professionals, group practicalities (content, recruitment, frequency, incentives, and limiting additional burden to staff), and their perceived value across stakeholders [12]. 

There is good evidence on the effectiveness of peer support interventions for people living with NCDs from stable or high income countries (HICs) [13–16]. Studies from HICs have suggested the following features were key to effectiveness: a duration of at least three to six months [17, 18]​, selecting participants with poorer clinical outcome measures e.g. higher HbA1c baseline measures [17, 19]​, a focus on behavioural and affective strategies [20, 21]​, exclusive provision of non-medical support [22], ensuring sensible selection of facilitators, and providing high-quality training [21]. 

However, evidence on peer support for NCDs from low- and middle-income countries (LMICs) and humanitarian settings is more limited. To the best of our knowledge, the sole study from a humanitarian context focussed on Palestinian refugees living with diabetes in Jordan [23]. Although this study showed an improvement in HbA1c, it did not have a control group and did not include people with hypertension. Thus, there is a significant gap on effective, acceptable, context adapted Peer Support Groups (PSGs) for people living with hypertension and or diabetes in humanitarian settings.

NCDs are of particular concern in Lebanon, which hosts almost 1.5 million Syrian refugees since the onset of the Syrian crisis in 2011 [24]. Diabetes and hypertension and their complications (including cardiovascular disease) are among the most common NCD morbidities affecting both refugee and host populations in Lebanon. They often coexist and are amenable to a primary level public health approach. Despite the Ministry of Public Health (MoPH) achieving some success in addressing the NCD burden in Lebanon, such as strengthening primary care within the public health system, major gaps in access, quality and consistency of NCD primary care remain [25]. An assessment of NCD care delivered by humanitarian actors in Lebanon highlighted that people living with NCDs experience a significant burden of care, drawing on their family and community networks to navigate a pluralistic health care system. Continuity at primary care level, information and referral systems, and medicine and equipment supply chains all require further strengthening [26]. 

In response to this, in 2021, the Danish Red Cross (DRC) and World Diabetes Foundation initiated the four-year *Bridging the Gap* project, in collaboration with the Lebanese Red Cross (LRC) and the Lebanese Ministry of Public Health. The project aimed to build a more coherent system of NCD prevention, care and support for Syrian refugees and Lebanese host communities, with a focus on capacity-building at health centre and community levels. The project had three main pillars: (1) community-level prevention; (2) access to healthcare; and (3) advocacy, research and partner engagement. The project employed two complementary implementation modalities: a comprehensive integrated model of prevention, screening, and care for NCDs provided in four target LRC primary healthcare centres (PHCs); and a lighter, community-based approach to NCDs, through community sensitisation, including psychosocial support, in eight locations where LRC’s mobile medical units and social workers were active. The integrated PHC model also included PSGs for people living with diabetes and hypertension in the four target centres. Youth-focused approaches to build NCD awareness and promote healthy living practices were included as components in both models, targeting five locations.

The design and implementation of *Bridging the Gap’s* NCD PSG component provides an opportunity to evaluate peer support approaches as part of NCD care in humanitarian settings. To learn lessons that could support humanitarian actors and the Lebanese health system in implementing and scaling up PSGs for NCDs in Lebanon and in other humanitarian settings, we conducted a mixed methods implementation study, including both qualitative and quantitative components [12]. The quantitative findings are presented here, and the qualitative findings will be reported in a separate paper.

## Methods

### Objectives

Our overall aim was to evaluate the implementation and impact of PSGs for Syrian and Lebanese host populations living with hypertension and/or diabetes (DM/HTN), implemented as part of an integrated NCD care model, within four target LRC-supported primary care centres in the humanitarian setting of Lebanon. Specifically, our objectives were to: (1) evaluate the reach of the PSGs; (2) evaluate the association of PSG with patient- reported outcomes; and (3) evaluate the association of PSGs with clinical outcomes (blood pressure, Hba1c and BMI).

### Design and settings

#### Study design

**Fig. 1:**
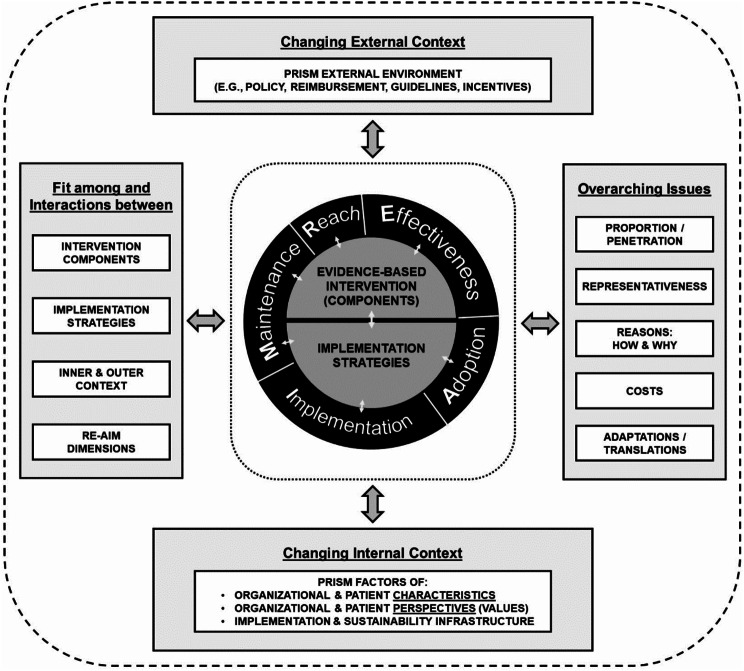
Re-AIM/PRISM Framework [25]

The overall study (including the qualitative component) was guided by the RE-AIM/PRISM implementation research framework (Fig. 1), which explores the characteristics of an intervention as well as the contexts under which an intervention is promoted or inhibited. The framework was used to inform the study design (e.g. for identifying relevant outcomes and data to be collected) and to guide the analysis and reporting [27, 28]. ​ Although there is no universally “correct” or singular framework for implementation research, the selection of a framework is usually guided by the research objectives. The RE-AIM/PRISM framework was chosen for this study due to its dual focus on intervention-specific outcomes and contextual factors that influence implementation success [29, 30]. Furthermore, the authors have prior experience using the RE-AIM/PRISM framework in conflict settings, demonstrating its relevance and feasibility for evaluating interventions in complex humanitarian contexts​ [31]. 

The quantitative component reported in this paper used a before-after study design, and included a control group for clinical outcomes. This findings are reported in accordance with the STROBE (Strengthening the Reporting of Observational Studies in Epidemiology) guidelines [32]. 

#### Setting: study sites

PSGs were implemented in four PHCs as part of the Bridging the Gap project and all were included in this evaluation. These four PHCs are located in Baalbek, Qab Elias, Jal El Dib, and Tripoli.

### Eligibility criteria

#### Patient recruitment

Participants were eligible to join the study if they had diabetes and/or hypertension, were 40 years or older and enrolled in any of the four LRC-supported PHCs. Recruitment was undertaken by trained LRC social workers via psycho-education sessions in waiting rooms of the participating health centres. Patients were approached as they visited the PHC and consented to either join a PSG or to have their data used as part of the control group. The term “vulnerable” in the context of this study refers to socio-economically disadvantaged Lebanese host population.

### PSG participants

The PSG intervention started in December 2022. Due to logistical constraints, the intervention was carried out in two waves. The first wave was implemented from December 2022 to July 2023, and the second wave was implemented from July 2023 to January 2024. PSGs participants were recruited and consented from May 2022 on a rolling basis. Patients were eligible to join PSG if they met the study eligibility criteria and were willing to commit to the PSGs. Potential participants were self-selecting i.e. interested patients signed up to participate in PSGs. More people were interested in joining PSGs than places available, therefore participants were further selected using clinical severity criteria. These included most recent or baseline HbA1c value greater than 48 mmol/mol (6.5%) or blood pressure measurement greater than 160/100 mmHg. The LRC team tried to ensure, as much as possible, representation of groups with members of both sexes and different nationalities in its selection. Based on these criteria, the social workers at each centre put the groups together based on their knowledge of the local context, the characteristics of potential participants and acceptability.

A total of 10–12 participants per PSG were selected with two groups running simultaneously at each centre, separated by gender and/or nationality at each facility depending on the local context and acceptability.

### Control group

To select the control group for the clinical outcomes, we used data collected from January 2023 to January 2024. Patients not participating in PSG were included in the control group using the same inclusion criteria for the PSG and had clinical outcome measures recorded in at least two visits with the first measure taken as baseline and the second measure as follow-up. For the first wave (wave 1), we included patients with baseline visit between January and March 2023 and a follow-up visit at 6 months or more. For the second wave (wave 2) we included patients with baseline visit between July and September 2023 and a follow-up visit at 3 months or more for HTN patients or 1 month or more for DM patients. While the plan was to enrol controls with a baseline and follow-up at approximately the same time as those enrolled in the PSGs, due to practical and logistical difficulties (data not available for the control group), the time frame for follow-up had to be adapted.

### Intervention description

#### PSG intervention design

**Fig. 2:**
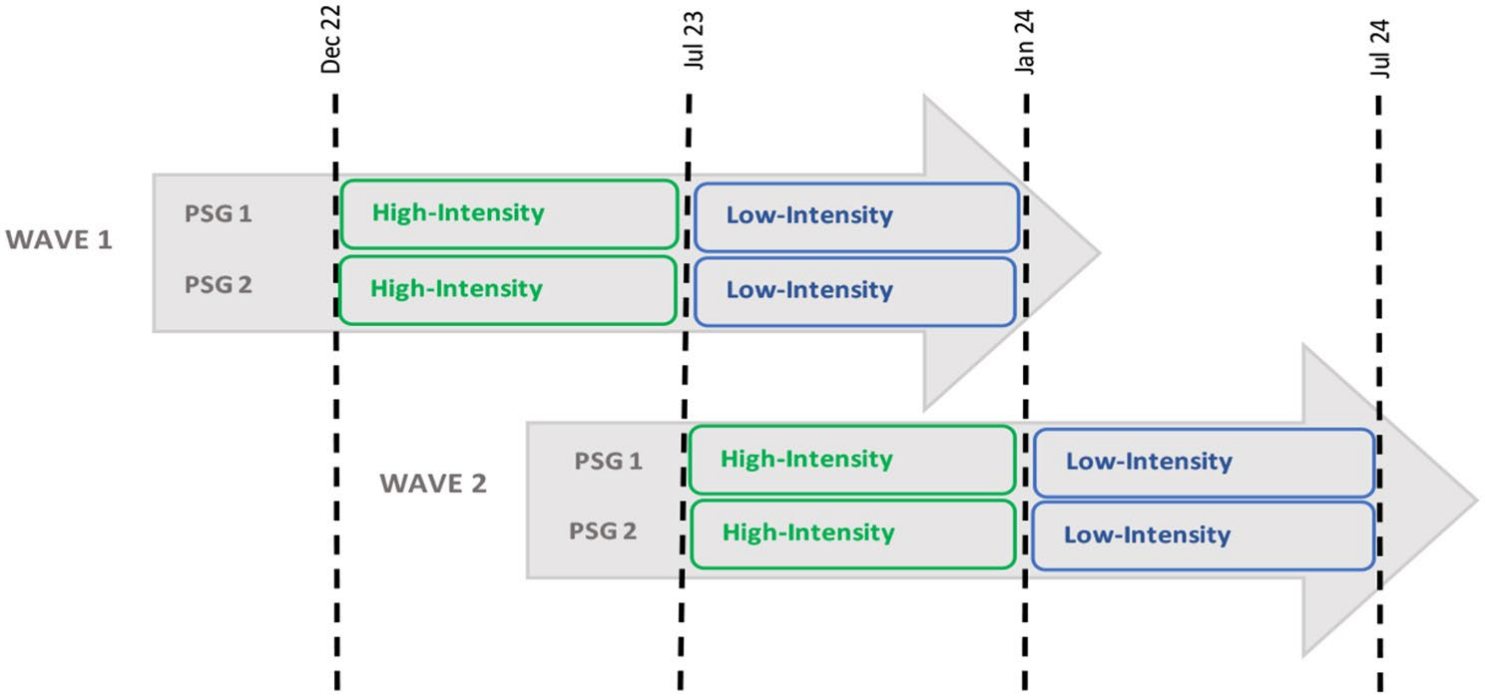
PSGs as implemented in each of the four target PHCs

The PSGs were facilitated by LRC social workers who were based at each study site and usually engaged in other activities. They received specific training based on a bespoke manual designed by LRC. Each PSG lasted for one year and was split into two parts (Fig. 2). The first six months was a *high-intensity* period facilitated by the social worker, supported by a volunteer and an LRC nurse. It consisted of bi-weekly meetings at an accessible location – either at the PHC, or in a building owned by the municipality. Months six to twelve comprised a *low-intensity* period where participants were encouraged to continue meeting voluntarily. LRC provided logistical support during this period but did not facilitate the meetings. Here, we only report only the evaluation of the high-intensity component.

### PSG content and implementation

PSG sessions during the *high-intensity* period were structured around themes of patient empowerment, diabetes, blood pressure, healthy eating, problem solving, stress management, physical activity, medication, mental activity, and social support. The content was based on LRC’s experience of PSGs for psychosocial support and was developed by a consultant in close collaboration with the LRC medical and psychosocial support teams. Culturally relevant patient education materials were newly developed by LRC and DRC to support the social workers and nurses in facilitating the knowledge-focused parts. The facilitating social workers and nurses received a three-day training course on DM,HTN and the course manual. Medical professionals supported the groups, according to the participants’ needs. The meeting locations were selected together with the participants to ensure accessibility, acceptability, and anonymity. Along with the face-to-face group meetings, the social worker also established a participant-only WhatsApp group, which the participants could use as desired. The *low-intensity* period consisted of unstructured and voluntary meetings based on the participants’ needs and desires.

#### Alignment with global good practice

Building on global best practice, the PSG design incorporated the following features: duration of six months (*high-intensity period*), targeted patients with higher clinical baseline measures, providing dedicated training and encouraging the facilitators to play an active role [17, 19, 21, 33]. The potential role of the group’s social network was not directly included in the design [23]. All other NCD-related interventions were the same for patients participating in the PSG and those in the control group.

### Outcomes

The study outcomes were defined using a theory of change co-designed by implementors and academic researchers, and informed by a literature review, the RE-AIM/PRISM framework [27, 34], implementation outcomes [35], and LRC’s experiences with psychosocial support PSGs. The selected outcomes featured service and patient outcomes as well as implementation outcomes as distinct measures [35]. 

The main outcomes of interest were:

1. Reach.

- % of approached patients who agreed to participate in PSGs​.

2. Patient reported outcome measures (PROMs) (before-after change in intervention).

- Patient quality of life (QoL).
- Behavioural factors (e.g., smoking, drinking, physical activity and treatment adherence).
- Patient centricity (i.e., quality of care meeting patients’ needs).

3. Clinical outcomes (comparison between intervention and control groups).

- Change in HbA1c.
- Change in systolic blood pressure (SBP).
- Change in BMI.

### Data collection tools and procedures

This study used the routinely collected programmatic data and administrative data. The patient PROMs were collected at baseline and at six-months by trained LRC volunteers for all PSG participants. The patient survey questions were extracted from different standard assessment tools. These included patients’ quality of life (WHOQOL-BREF) [36]​, person-centredness of care (CollaboRATE) [37]​, risk behaviours (WHO-STEPS) ​(World Health Organisation., 2005)​ and treatment adherence (MARS-5) [38]. ​ The survey was conducted in Arabic using validated tools. The CollaboRATE scale was translated through a comprehensive translation process by LRC recruited translators in an independent forward- and back-translation, as recommended by the tool originators. All tools were piloted before use. Clinical data were extracted from routine programmatic data. This data contained patients’ demographics and general characteristics (such as age, sex, comorbidities, nationality, education levels) and clinical measures (such as SBP, diastolic blood pressure (DBP), HbA1c, and BMI). Administrative data was part of the Bridging the Gap PSG monitoring and evaluation process. As per LRC’s routine care, blood pressure measurements were typically recorded at every patient visit, while HbA1c was measured every three to six months. All datasets were in Excel format and were imported into STATA version 17.0 for data merging, cleaning and analysis, using a unique identifier.

### Statistical analysis

#### Sample size

At the time of recruitment, the four centres had a total of 2023 registered DM/HTN patients (Qob Elias: 668, Tripoli: 686, Jal El Dib: 349, and Baalbek: 320). Due to resource and capacity issues, inclusion into the PSGs was limited to approximately 10 participants per PSG with two groups running simultaneously at each of the four centres in two successive waves (equating to 16 groups). Therefore, we planned to enrol 160 participants in total. For the control group, we selected all patients who fulfilled the inclusion criteria, who were not enrolled in a PSG, and for whom at least two clinical outcomes measurements were available (one at baseline and one at follow-up), recorded at approximately the same time as the PSGs took place. In practice, this timeframe had to be adapted according to data availability. Assuming at least one control was selected per PSG participant, a sample of 320 (160 patients in the PSGs and 160 controls) would give more than 80% power to observe a difference of 7.5 mmHg change in SBP (with a standard deviation (SD) in each group of 23 mmHg) and 0.55% change in HbA1c (SD in each group of 1.7%) between the PSG and control groups. However, the planned analysis incorporated adjustment for baseline measures of SBP and HbA1c, which are known to be highly correlated within individuals. Hence, this increased power and allowed for loss to follow-up. We calculated that if we included three controls per PSG participant, that is a sample of 640 (160 in the PSGs and 480 controls), this would give more than 95% power to detect the differences described above within each wave. To maximize power, therefore, we decided to include all NCD patients attending the target clinics (and not enrolled in a PSG) that fulfilled the inclusion criteria and had the relevant clinical outcome measures recorded within an appropriate time window.

#### Data management and analysis

Data were checked for participant eligibility, possible errors and missing data using frequencies, histograms and range. Descriptive statistics were summarized using frequencies and proportions for categorical variables, while mean and SD or medians and inter-quartile ranges were used to summarize continuous data, depending on the distribution of continuous variables.

We performed a before-after analysis of PROMs for all patients who completed the PSG sessions. T-tests were used to analyse the differences from baseline in PROMs, and p-values for the changes were reported.

To assess the impact on participants’ quality of life, we utilized the WHOQOL-BREF tool, which measures four domains (physical, psychological, social, and environmental) and an overall QoL score. Changes in scores were evaluated to determine both statistical and clinical relevance.

While the concept of Minimal Clinically Important Difference (MCID) is valuable for interpreting the clinical significance of observed changes, to the best of our knowledge, universally accepted MCID thresholds for the WHOQOL-BREF are not well-established in our study population (NCDs in conflict settings). Therefore, in the absence of universally applicable MCID values, we interpreted changes in QoL scores by considering both statistical significance and the potential clinical relevance, informed by existing literature [39]. 

Based on existing literature, MCID changes of **0.2–0.4 points on a 5-point Likert scale** (equivalent to **4–8 points on a 100-point scale**) are considered clinically meaningful for the WHOQOL-BREF overall QoL score and domains.

The original intention was to combine data from the two PSG waves, and also present separate descriptions of the data by wave. However, there were clear and substantial differences in both the characteristics and outcomes across the two waves as well as seasonal changes across the year and differences in the length of follow-up in the controls between waves 1 and 2. Therefore, results are presented separately by wave, and a secondary analysis combining the two waves for the main outcomes of SBP and HbA1c is also reported. This approach limits power for the clinical outcomes, although this was mitigated to an extent by the increased number of controls selected, together with the analysis incorporating the correlation in the baseline and follow-up measurements.

The trend and pattern of clinical outcomes for PSG participants and control group, from baseline to 6 months after introduction of PSGs, were assessed. Descriptive trend analyses were undertaken to compare the differences in the change of outcomes over time between the PSG and control groups.

To assess the association between implementation of the PSG strategy and the clinical outcomes (SBP HbA1c and BMI change), analysis of covariance (ANCOVA) models were used, adjusting for the baseline values of the outcome being analysed (baseline SBP and baseline HbA1c, respectively). The ANCOVA models were also adjusted for the potential confounders of age and sex. The models are presented in both unadjusted and adjusted form, using differences in the change between groups as the effect estimate, together with 95% CIs.

## Results

### Reach

445 patients were approached for enrolment in wave 1 PSG, of these, 259 (58%) consented to participate in the PSG program, and 81 (18%) were ultimately enrolled. In wave 2, 169 patients were approached, of whom 92 (54%) consented to participate in the PSG program, and almost everyone (91 (54%)) was ultimately enrolled. (Table 1)

### Characteristics of participants

Most wave 1 PSG participants were married (84%), Lebanese (95%), and female (80%). Additionally, 52% were diagnosed with both HTN and DM, and 49% of participants had obesity. A total of 13 participants (16%) dropped out. Drop-outs had similar characteristics to those who completed follow-up in the PSG group.

Similar to wave 1, the majority of wave 2 PSG participants were married (82%), Lebanese (87%), diagnosed with both HTN and DM (57%) and overweight (49%), although there was a more even split between females (52%) and males. The 24 (26%) participants who dropped out had similar characteristics to those completed follow-up in the PSG group.

**Table 1:** Characteristics of approached patients compared to consented patients​ and PSG participants in both waves

	Approached	Consented	Participated in PSG
	*n* (column %)	*n* (row %)	*n* (row %)
**WAVE 1**			
**Total**	445	259 (58)	81 (18)
**Diagnosis**			
Diabetes only	110 (25)	66 (60)	13 (12)
Hypertension only	175 (39)	94 (54)	26 (15)
Both Diabetes and Hypertension	160 (36)	99 (62)	42 (26)
**Nationality**			
Lebanese	417 (94)	245 (59)	77 (18)
Syrian	28 (6)	14 (50)	4 (14)
**Facility**			
Qab Elias	237 (53)	106 (45)	20 (8)
Baalback	72 (16)	56 (78)	19 (26)
Jal El Dib	50 (11)	35 (70)	22 (44)
Tripoli	86 (19)	62 (72)	20 (23)
**WAVE 2**			
**Total**	169	92 (54)	91 (54)
**Diagnosis**			
Diabetes only	41 (24)	19 (46)	19 (46)
Hypertension only	45 (27)	21 (47)	20 (44)
Both Diabetes and Hypertension	82 (48)	52 (63)	52 (63)
Other	1 (1)		
**Nationality**			
Lebanese	154 (91)	80 (52)	79 (51)
Syrian	12 (7)	10 (83)	10 (83)
Palestinian	3 (2)	2 (67)	2 (67)
**Facility**			
Qab Elias	53 (31)	20 (38)	19 (36)
Baalback	32 (19)	24 (75)	24 (75)
Jal El Dib	49 (29)	23 (47)	23 (47)
Tripoli	35 (21)	25 (71)	25 (71)

## PROMs: quality of life, treatment adherence patient centredness and behavioural risk factors: comparison before and after psg

### Wave 1

In wave 1, we found improvement in most of the PROMs, with five outcomes showing some statistical evidence (*p* ≤ 005) of a clinically meaningful improvement. The QoL score increased by 0.4 (SD: 1.0) points. There was also a 5.8 (SD:16.9) point increase in the physical QoL domain, and a 4.6 (SD:18.4) point increase in the environmental domain. The percentage of people who walked or used a bicycle for at least 10 min continuously for travel also increased by 15% after PSG participation. Finally, we also found some statistical evidence of an increase in patient centeredness (Table 2).

**Table 2:** Change in QoL, treatment adherence and patient centredness after W1 PSG sessions

	Overall Difference	*P*-value for difference (H0: diff = 0)
General QoL score, mean (SD)	**0.4 (1.0)**	**< 0.001**
General health score, mean (SD)	0.2 (1.4)	0.202
Physical domain score, mean (SD)	**5.8 (16.9)**	**0.007**
Psychological domain score, mean (SD)	3.3 (18.6)	0.163
Social domain score, mean (SD)	1.8 (27.0)	0.594
Environmental domain score, mean (SD)	**4.6 (18.4)**	**0.05**
**Medication adherence**		
MARS-5 score, mean (SD)	-0.1(3.7)	0.8926
MARS-5 score > 19, (%)	2	0.8331
**Patient centredness**		
CollaboRATE score > 8, (%)	**29**	**< 0.001**
Smoking, (%)	3	0.718
Intend to reduce smoking in foreseeable future, (%)	19	0.159
Drinking, (%)	0	1
Intend to reduce drinking in foreseeable future, (%)	25	0.285
Eating fruits at least once day/week, (%)	-2	0.698
Eating vegetables at least once day/week, (%)	-2	0.559
Intend to change diet in foreseeable future, (%)	11	0.208
Walk or use bicycle for at least 10 min continuously for travel, (%)	**15**	**0.015**
Intend to increase amount of physical activity in foreseeable future, (%)	2	0.861

### Wave 2

In wave 2, we also found some statistical evidence of improvement in some outcomes. The QoL social domain score increased 8.5 (SD 24.9) points while the adherence score improved by 1.2 (SD: 3.1) points and overall, the percentage of participants who were adherent to medication increased by 13% after PSG sessions. (Table 3)

**Table 3:** Change in QoL, treatment adherence and patient centredness after W2 PSG sessions

	Overall Difference	*P*-value for difference (H0: diff = 0)
General QoL score, mean (SD)	0.3 (1.1)	0.055
General health score, mean (SD)	-0.1 (1.1)	0.729
Physical domain score, mean (SD)	-0.2 (18.0)	0.939
Psychological domain score, mean (SD)	-1.1 (20.1)	0.687
Social domain score, mean (SD)	**8.5 (24.9)**	**0.011**
Environmental domain score, mean (SD)	-0.9 (20.6)	0.738
**Medication adherence**		
MARS-5 score, mean (SD)	**1.2 (3.1)**	**0.004**
MARS-5 score > 19, (%)	**13**	**0.041**
**Patient centredness**		
CollaboRATE score > 8, (%)	0	1
Smoking, (%)	4	0.713
Intend to reduce smoking in foreseeable future	16	0.189
Drinking, (%)	-8	0.19
Intend to reduce drinking in foreseeable future, (%)	14	0.585
Eating fruits at least once day/week, (%)	3	0.464
Eating vegetables at least once day/week, (%)	5	0.079
Intend to change diet in foreseeable future, (%)	-3	0.783
Walk or use bicycle for at least 10 min continuously for travel, (%)	15	0.07
Intend to increase amount of physical activity in foreseeable future, (%)	7	0.442

### Clinical outcomes

For the clinical outcomes, we compared the changes observed before and after the PSG intervention with the changes during similar periods in the control groups.

### Control group participants selection

A total of 1389 patients had outcome measures recorded in both the baseline and a follow-up visit. Of these, 301 had a baseline between January and March 2023 (wave 1) and a follow-up visit at 6 months or more. These made-up wave 1 controls. Another 327 patients had a baseline between July and September 2023 (wave 2) and a follow-up visit at 3 months or more for hypertensives and 1 month or more for diabetics. These patients made up wave 2 controls.

### Baseline characteristics of the intervention and control groups

Some differences were observed in the baseline characteristics of the intervention and control groups. For example, in wave 1, in the control group there was a higher proportion of male patients, and in both waves, we observed a higher proportion of patients with HTN in the control groups. Also, we observed that patients in the control group achieved better clinical control for both diabetes and hypertension. (Table 4)

**Table 4:** Demographic characteristics of PSG and control group for both waves

	WAVE 1			WAVE 2		
	PSG Participants		Control group	PSG Participants		Control group
	All	Drop-outs		All	Drop-outs	
	**n (%)**	**n (%)**	**n (%)**	**n (%)**	**n (%)**	**n (%)**
**Total**	81	13	301	91	24	327
**Diagnosis**						
Diabetes	13 (16.1)	0 (0)	32 (10.6)	19 (20.9)	5 (20.8)	60 (18.4)
Hypertension	26 (32.1)	5 (38.5)	229 (76.1)	20 (22)	2 (8.3)	186 (56.9)
Both	42 (51.8)	8 (61.5)	40 (13.3)	52 (57.1)	17 (70.8)	81 (24.8)
**Diabetes Control (< 7%)**						
Yes	27 (49.1)	5 (62.5)	45 (62.5)	27 (38.0)	7 (31.8)	67 (47.5)
No	28 (50.9)	3 (37.5)	27 (37.5)	44 (62.0)	15 (68.2)	74 (52.5)
**BP Control (< 140/90)**						
Yes	21 (30.9)	3 (23.1)	156 (58.0)	37 (51.4)	9 (47.4)	172 (64.4)
No	47 (69.1)	10 (76.9)	113 (42.0)	35 (48.6)	10 (52.6)	95 (35.6)
Age*	61.0 (8.2)	63 (8.8)	62.0 (9.6)	60.9 (8.9)	62.1 (9.5)	63.2 (9.9)
Age Range	44–78	47–77	40–94	43–88	45–88	40–88
**Sex**						
Male	16 (19.8)	4 (30.8)	159 (52.8)	44 (48.3)	12 (50)	144 (44)
Female	65 (80.2)	9 (69.2)	142 (47.2)	47 (51.7)	12 (50)	183 (56)
**BMI**						
Underweight	1 (1.2)	0 (0)	0 (0)	0 (0)	0 (0)	3 (0.9)
Healthy	9 (11.1)	1 (7.7)	49 (16.3)	14 (15.4)	3 (12.5)	51 (15.6)
Overweight	31 (38.3)	8 (61.5)	126 (41.9)	45 (49.5)	13 (54.2)	113 (34.6)
Obese	40 (49.4)	4 (30.8)	124 (41.2)	32 (35.2)	8 (33.3)	123 (37.6)
Missing	0 (0)	0 (0)	2 (0.7)	0 (0)	0 (0)	37 (11.3)
**Marital status**						
Married	68 (83.9)	10 (76.9)		75 (82.4)	17 (70.8)	
Single	4 (4.9)	2 (15.4)		5 (5.5)	2 (8.3)	
Widowed	1 (1.2)	1 (7.7)		7 (7.7)	3 (12.5)	
Divorced	8 (9.9)	0 (0)		4 (4.4)	2 (8.3)	
**Nationality**						
Lebanese	77 (95.1)	13 (100)	286 (95)	79 (86.8)	23 (95.8)	316 (96.6)
Syrian	4 (4.9)	0 (0)	12 (4)	10 (11)	1 (4.2)	9 (2.7)
Palestinian	0 (0)	0 (0)	2 (0.7)	2 (2.2)	0 (0)	0 (0)
Other	0 (0)	0 (0)	1 (0.3)	0 (0)	0 (0)	2 (0.6)
**Facility**						
Qab Elias	20 (24.7)	2 (15.4)	145 (48.2)	19 (20.9)	5 (20.8)	65 (24.2)
Baalback	19 (23.5)	3 (23.1)	132 (92)	24 (26.4)	8 (33.3)	98 (30)
Jal El Dib	22 (27.2)	7 (53.8)	7 (2.3)	23 (25.3)	6 (25)	123 (37.6)
Tripoli	20 (24.7)	1 (7.7)	17 (5.7)	25 (27.5)	5 (20.8)	27 (8.3)

*Mean (SD)

## Changes in clinical outcomes between psg and control group

### Wave 1

#### SBP

We observed a decrease in SBP in both PSG (Mean change − 5.6mmHg; SD 22.6mmHg) and control groups (Mean change − 0.9mmHg; SD 17.2mmHg); the decrease was larger (-4.7mmHg) in the PSG group. (Table 5). However, in the adjusted analysis (by age, sex and baseline SBP), we did not find strong statistical evidence of a larger decrease (-0.7mmHg [95% CI -5.08 to 3.69mmHg]) in the PSG group (Table 6).

#### HBA1C

A very small increase in HbA1c was observed for both groups although the increase was slightly lower in the PSG (0.01%; SD 0.8%) in comparison to the control arm (0.03%; SD 1.2%) (Table 5). In the adjusted analysis (by age, sex, and baseline HbA1c), we did not find strong statistical evidence of a decrease in the PSG groups (Table 6).

#### BMI

We observed an increase in BMI in PSG (0.5 Kg/m^2^; SD 2.8 Kg/m^2^) while no change in control group (0.0 Kg/m^2^; SD 1.2 Kg/m^2^). (Table 5). After adjusting for age, sex and baseline BMI we observed some statistical evidence of a small increase (0.6 Kg/m^2^) in BMI in PSG participants. (Table 6)

**Table 5:** Descriptive change in outcomes between W1 PSG and control group

	PSG (*n* = 68)									CONTROL (*n* = 301)									
	SBP (*n* = 68)			HbA1c (*n* = 68)			BMI (*N* = 68)			SBP (*n* = 301)			HbA1c (*n* = 72)			BMI (*n* = 261)			
	Baseline	Endline	Change	Baseline	Endline	Change	Baseline	Endline	Change	Baseline	Endline	Change	Baseline	Endline	Change	Baseline	Endline	Change	
**Average**	138.8 (19.2)	133.2 (16.5)	-5.6 (22.6)	7.0 (1.8)	7.0 (1.6)	0.01 (0.8)	31.1 (6.1)	31.6 (5.6)	0.5 (2.8)	133.0 (15.9)	132.1 (17.4)	-0.9 (17.2)	7.3 (1.7)	7.3 (1.7)	0.03 (1.2)	29.5 (5.0)	29.5 (5.2)	0.0 (1.2)	
**Sex**																			
Male	146.7 (18.7)	131.6 (14.2)	-15.1 (23.1)	7.6 (2.0)	7.4 (1.8)	-0.2 (1.1)	27.0 (3.2)	30.4 (4.2)	3.4 (4.8)	135.1 (16.0)	134.3 (17.4)	-0.8 (17.1)	7.4 (1.7)	7.2 (1.5)	-0.2 (1.4)	28.6 (4.4)	28.5 (4.5)	-0.0 (0.8)	
Female	137.1 (19.0)	133.6 (17.0)	-3.5 (22.2)	6.8 (1.7)	6.9 (1.6)	0.04 (0.8)	32.0 (6.7)	31.9 (5.9)	-0.1 (1.7)	130.7 (15.5)	129.6 (17.0)	-1.1 (17.4)	7.1 (1.8)	7.4 (2.1)	0.3 (0.7)	30.5 (5.5)	30.7 (5.7)	0.1 (1.5)	
**Diagnosis**																			
Diabetes	140.2 (17.3)	131.3 (16.3)	-8.7 (19.2)	7.5 (1.4)	7.7 (1.4)	0.2 (0.7)	29.8 (5.6)	31.0 (5.1)	1.2 (4.6)	128.1 (16.5)	132.1 (19.2)	4.1 (16.2)	7.6 (2.1)	7.4 (1.9)	-0.2 (1.6)	28.3 (5.3)	27.7 (5.8)	-0.5 (1.8)	
Hypertension	139.5 (16.3)	134.1 (13.1)	-5.4 (20.2)	5.6 (0.4)	5.7 (0.5)	0.2 (0.4)	32.8 (6.5)	32.7 (6.3)	-0.1 (1.7)	132.7 (15.4)	131.9 (17.3)	-0.8 (16.9)	N/A	N/A	N/A	29.4 (5.0)	29.5 (5.1)	0.1 (1.2)	
Both	137.9 (21.8)	133.4 (18.7)	-4.5 (25.5)	7.6 (1.9)	7.4 (1.8)	-0.2 (1.0)	30.5 (5.8)	31.2 (5.5)	0.7 (2.5)	139.1 (16.5)	133.5 (16.7)	-5.6 (18.7)	7.0 (1.3)	7.2 (1.7)	0.2 (0.8)	30.8 (5.4)	30.7 (5.4)	-0.1 (0.9)	
**BMI**																			
Underweight																			
Healthy	141.2 (18.1)	128.1 (11.9)	-13.1 (20.1)	6.5 (0.7)	6.8 (0.9)	0.3 (0.6)				129.5 (16.3)	128.2 (15.2)	-1.3 (17.2)	8.5 (1.9)	8.4 (1.9)	-0.04 (0.8)				
Overweight	140.0 (17.6)	131.1 (13.5)	-8.9 (19.3)	7.1 (1.7)	7.0 (1.6)	-0.1 (0.8)				131.0 (16.0)	131.1 (17.6)	0.1 (15.4)	7.3 (1.9)	7.1 (1.8)	-0.1 (1.5)				
Obese	138.9 (19.4)	135.9 (19.0)	-2.9 (24.2)	7.0 (2.0)	7.0 (1.8)	0.01 (0.9)				136.0 (14.7)	136.5 (7.8)	-1.3 (18.7)	7.1 (1.4)	7.2 (1.7)	0.1 (1.1)				

**Table 6:** Overall crude and adjusted association between W1 PSG and change in SBP and HbA1c

	Change in SBP (*n* = 369)		Change in HbA1c (*n* = 140)		Change in BMI (*N* = 329)	
	Beta (95%CI)	aBeta (95%CI)	Beta (95%CI)	aBeta (95%CI)	Beta (95%CI)	aBeta (95%CI)
**Intervention**						
Control	-	-	-	-	-	-
PSG	-4.66 [-9.50 to 0.18]	-0.70 [-5.08 to 3.69]	-0.02 [-0.37 to 0.33]	-0.35 [-0.71 to 0.01]	**0.49 [0.04 to 0.94]**	**0.63 [0.16 to 1.09]**
Baseline SBP (mmHg)	**-0.58 [-0.67 to -0.48]**	**-0.58 [-0.68 to -0.48]**				
Baseline HbA1c (%)			**-0.20 [-0.29 to -0.11]**	**-0.21 [-0.30 to -0.12]**		
**Sex**						
Male	-	-	-	-	-	-
Female	-0.01 [-3.79 to 3.78]	-1.91 [-5.32 to 1.49]	0.35 [0.00 to 0.71]	**0.40 [0.03 to 0.76]**	-0.16 [-0.52 to 0.21]	-0.18 [-0.57 to 0.20]
**Age/10 (years)**	-0.61 [-2.62 to 1.14]	-0.48 [-2.21 to 1.26]	0.20 [-0.0 to 0.41]	**0.29 [0.10 to 0.49]**	-0.05 [-0.25 to 0.14]	-0.11 [-0.31 to 0.08]
**Baseline BMI**						
Healthy					-	-
Underweight					omitted	omitted
Overweight					-0.52 [-1.05 to 0.01]	**-0.56 [-1.09 to -0.03]**
Obese					**-0.90 [-1.43 to -0.38]**	**-0.98 [-1.52 to -0.44]**

### Wave 2

#### SBP

Unlike in wave 1, we observed an increase in SBP in both PSG (Mean change 6.8mmHg; SD 16.9mmHg) and control group (Mean change 5.1mmHg; SD 18.0mmHg) among wave 2 participants. (Table 7). This observed increase in SBP over time in both groups was related to higher SBP measures recorded during endline period (winter) and lower SBP measurements during baseline (summer) (Figure S1). After adjusting by sex, age and baseline SBP we did not find strong statistical evidence of a change of SBP among the two groups (Table 8).

#### HbA1c

A very small drop in HbA1c was observed for the PSG group (− 0.1%; SD 0.8%) while no change was observed in the control group (change 0.0%; SD 1.5%) (Table 7). When adjusting by sex, age and baseline HbA1c, we did not find strong statistical evidence for sthe change in HbA1c (Table 8).

#### BMI

We observed a small increase in BMI in both PSG (0.3 Kg/m^2^; SD 2.4 Kg/m^2^) and control groups (0.1 Kg/m^2^; SD 0.7 Kg/m^2^) (Table 7). However, when adjusting for age, sex and baseline BMI, we did not find strong difference among the two groups. (Table 8)

**Table 7:** Descriptive change in outcomes between W2 PSG and control group

	PSG (*N* = 67)									CONTROL (*N* = 327)								
	SBP (*n* = 67)			HbA1c (*n* = 67)			BMI (*n* = 67)			SBP (*n* = 324)			HbA1c (*n* = 141)			BMI (*n* = 151)		
	Baseline	Endline	Change	Baseline	Endline	Change	Baseline	Endline	Change	Baseline	Endline	Change	Baseline	Endline	Change	Baseline	Endline	Change
**Average**	133.4 (20.8)	140.2 (23.3)	6.8 (16.9)	7.2 (1.7)	7.1 (1.5)	-0.1 (0.8)	29.0 (4.7)	29.3 (4.7)	0.3 (2.4)	130.4 (19.0)	135.6 (17.2)	5.1 (18.0)	7.6 (1.8)	7.3 (1.7)	**-0.3 (1.5)**	30.1 (6.3)	30.1 (6.4)	0.1 (0.7)
**Sex**																		
Male	135.4 (19.0)	144.7 (24.7)	9.3 (18.3)	7.3 (1.6)	7.2 (1.6)	-0.1 (0.8)	28.5 (4.8)	29.2 (4.8)	0.7 (2.2)	131.2 (19.5)	136.8 (17.7)	5.6 (17.5)	7.8 (1.8)	7.5 (1.9)	-0.2 (1.3)	28.8 (5.3)	28.9 (5.2)	0.1 (0.6)
Female	131.5 (22.5)	136.1 (21.5)	4.5 (15.1)	7.2 (1.8)	7.1 (1.5)	-0.1 (0.7)	29.5 (4.6)	29.5 (4.8)	-0.1 (2.5)	129.7 (18.7)	134.5 (16.8)	4.7 (18.4)	7.4 (1.9)	7.1 (1.4)	-0.3 (1.6)	31.0 (6.9)	31.0 (7.0)	0.0 (0.8)
**Diagnosis**																		
Diabetes	128.1 (20.4)	140.1 (19.1)	11.9 (15.0)	8.2 (1.7)	8.1 (1.6)	-0.1 (0.8)	30.0 (5.7)	30.4 (6.3)	0.4 (1.6)	123.6 (18.6)	131.7 (18.5)	8.0 (13.9)	7.9 (2.0)	7.7 (2.0)	-0.2 (1.5)	30.8 (6.0)	30.8 (5.9)	0.0 (0.2)
Hypertension	133.7 (17.5)	138.5 (15.6)	4.8(14.8)	5.9 (0.8)	5.9 (0.7)	-0.1 (0.4)	27.9 (4.5)	28.2 (4.9)	0.3 (3.5)	132.2 (18.1)	135.2 (16.7)	3.0 (17.9)	N/A	N/A	N/A	29.8 (6.4)	29.8 (6.4)	0.0 (0.7)
Both	135.3 (22.6)	141.1 (28.1)	5.8 (18.2)	7.5 (1.6)	7.4 (1.4)	-0.1 (0.9)	29.3 (4.3)	29.5 (3.9)	0.2 (2.0)	131.3 (20.6)	139.2 (17.0)	7.9 (20.2)	7.4 (1.7)	7.1 (1.4)	-0.3 (1.5)	32.6 (5.8)	32.7 (5.8)	0.1 (0.4)
**BMI**																		
Underweight	-	-	-	-	-	-				101.0 (4.6)	111.3 (9.1)	10.3 (6.4)	-	-	-			
Healthy	125.9 (21.6)	135.8 (26.6)	9.9 (24.4)	7.0 (1.9)	6.9 (2.0)	-0.1 (0.6)				129.3 (18.4)	135.4 (17.7)	6.1 (18.8)	7.9 (2.2)	7.7 (2.0)	-0.3 (0.9)			
Overweight	132.8(15.9)	141.1 (18.5)	9.5 (24.4)	6.9 (1.4)	6.9 (1.3)	0.0(0.7)				130.5 (19.8)	136.1 (17.3)	5.6 (17.2)	7.4 (1.7)	7.5 (1.8)	0.1 (1.3)			
Obese	138.3 (25.5)	140.4 (28.0)	1.8 (15.3)	7.8 (1.9)	7.6 (1.6)	-0.2 (0.9)				130.0 (19.5)	134.7 (16.7)	4.7 (19.0)	7.6 (1.6)	7.1 (1.2)	-0.5 (1.4)			

**Table 8:** Overall crude and adjusted association between PSG and change in SBP, HbA1c and BMI

	SBP (*n* = 393)		HbA1c (*n* = 208)		Change in BMI	
	Beta (95%CI)	aBeta (95%CI)	Beta (95%CI)	aBeta (95%CI)	Beta (95%CI)	aBeta (95%CI)
**Intervention**						
Control	-	-	-	-	-	-
PSG	1.68 [-3.01 to 6.38]	3.05 [-1.01 to 7.12]	0.20 [-0.18 to 0.58]	**0.07 [-0.27 to 0.41]**	0.25 [-0.17 to 0.66]	0.19 [-0.24 to 0.62]
Baseline SBP (mmHg)	**-0.47 [-0.54 to -0.39]**	**-0.47 [-0.55 to -0.39]**				
Baseline HbA1c (%)			**-0.34 [-0.43 to -0.25]**	**-0.34 [-0.43 to -0.25]**		
**Sex**						
Male	-	-	-	-	-	-
Female	-1.57 [-5.12 to 1.98]	-2.40 [-5.46 to 0.67]	-0.03 [-0.39 to 0.32]	-0.11 [-0.43 to 0.21]	-0.32 [-0.71 to 0.06]	-0.29 [-0.68 to 0.10]
**Age/10 (years)**	-0.81 [-2.61 to 1.00]	0.19 [-1.38 to 1.76]	0.07 [-0.12 to 0.26]	0.06 [-0.11 to 0.23]	0.02 [-0.22 to 0.19]	0.05 [-0.26 to 0.17]
**Baseline BMI**						
Healthy					-	-
Underweight					omitted	omitted
Overweight					-0.10 [-0.64 to 0.44]	-0.15 [-0.69 to 0.40]
Obese					-0.42 [-0.94 to 0.10]	-0.42 [-0.96 to 0.12]

## Discussion

To our knowledge, this is the first comprehensive evaluation of PSGs for people with hypertension and diabetes in humanitarian settings. Our study showed mixed results. In terms of reach, more than half of those approached consented to participate. The reasons for declining are likely multifaceted, potentially including the requirement to sign informed consent forms, the nature of the intervention, or other personal reasons related to work, distance, transportation cost or lack of time. From those who were enrolled, only 16% and 26% of participants did not complete the six-month period in the first and second waves, respectively. The main reasons for dropping out were work, transportation cost, loss of interest or participants or family member’s sickness.

Regarding the impact on PROMs, we observed an improvement in most of the outcomes, including overall quality of life (wave 1), physical quality of life (wave 1), social quality of life (wave 2), environmental quality of life (wave 1), adherence (wave 2), patient centeredness (wave 1), and exercise (wave 1).

The differences observed in the PROMs between wave 1 and wave 2 could be attributed to several factors. They may be related to the differences in the populations participating in each wave, differences in the way the intervention was delivered or in the individuals implementing it. Additionally, since we measured multiple outcomes, it is possible that some of the observed differences may be due to chance. Our ongoing qualitative study, to be published separately, may shed some light on these findings.

In terms of clinical outcomes, although we observed a marked improvement in SBP in wave 1, in the adjusted analysis there was no strong statistical evidence that this improvement was greater than that observed in the control group. One noteworthy aspect was the opposite trends in SBP for both the intervention and control group in the two waves (a decrease for both groups in wave 1 and an increase for both in wave 2). This may be explained by the well-known effects of temperature on SBP as wave 2 endline measurements coincided with the winter season [40]. Furthermore, shorter follow-up time might have impacted the results we observed, as evidence show that HbA1c improved with longer follw-up time for group-based interventions [41]. Overall, we did not find strong statistical evidence of an association between any of the clinical outcomes assessed once we adjusted for the baseline values, sex and age.

Results from our study are not consistent with the only other study evaluating the effect of a “Micro clinic Social Network” programme for diabetic Palestinian refugees in Jordan, Lebanon, West Bank and Gaza run by United Nations Relief and Works Agency (UNRWA), that showed an improvement on HbA1c. However, this study did not use a control group and it is not clear what other concurrent interventions were implemented beyond the education activities included in the programme.

### Strength and limitations

To our current knowledge, this is the first implementation study to evaluate a peer-support group intervention for people with hypertension and/or diabetes in humanitarian settings. Conducting research in unstable settings is inherently challenging, particularly for chronic conditions, which have historically been neglected in crisis response, and which require methodological approaches to account for their prolonged nature that are difficult to implement. The fact that this is the first comprehensive evaluation of PSGs for the two most common NCD conditions treated in humanitarian settings (hypertension and diabetes) underscores the magnitude of the challenge.

Among our study’s strengths we can highlight the use of a formal framework (RE-AIM) and a theory of change to inform the study design. Another crucial element was the strong partnership between the humanitarian and research organisations. This partnership was instrumental in designing a study that included relevant outcomes across the full spectrum, from PROMs to clinical ones, and in developing a methodological approach that used routinely collected programmatic and clinical data without disrupting the daily operations of the humanitarian organisations. Additionally, our use of a control group for clinical outcomes and multivariable adjustment represents another methodological strength.

Our study is not without limitations. Although all the patients who fulfilled inclusion criteria were invited to participate, only a small proportion ultimately joined the PSGs, which might have introduced some selection bias and limited the generalizability of our findings. In particular, a very low rate of refugees participated. For the evaluation of PROMs, we lacked a control group for comparison, limiting our ability to assess the changes more robustly. Furthermore, it is important to note the MCID changes considered clinically relevant were derived from stable and chronic disease populations in other populations, which might differ from the context of this study. Despite this limitation, these thresholds represent the only available benchmarks.

Regarding the clinical outcomes, although we included a control group and applied multivariable adjustments, the data available only included a small group of potential confounders. Given the non-randomised nature of the study, unmeasured and residual confounding remains a possibility. Additionally, although we aimed, as much as possible, to obtain data from the control group that coincides with the timing of baseline and endline measurement of the clinical outcomes in PSGs, for wave 2, and in particular for HbA1c, the interval was shorter in the control group. Our intervention group sampling was defined by logistical issues (in terms of feasibility for the humanitarian organisation) so we might not have had enough power to detect some real effects of PSGs. Finally, because we tested many outcomes, the likelihood of finding significant results by chance increases, leading to a potential inflation of false positives. Therefore, while some p-values may appear to show some statistical evidence of an effect, it is important to interpret these results cautiously.

### Implications for research and policy

PSGs is an intervention that is potentially important for humanitarian settings, and our studies will inform other organisations about its potential implementation and impact. In addition to our research activities, in the context of this partnership, we have already published a Peer Support Handbook that other humanitarian can use to implement such programmes (Resources | Partnering for Change (P4C) (humanitarianncdaction.org). As mentioned, a separate qualitative study will be published to explore factors influencing the programme’s implementation and capture the views of people with lived experiences.

Furthermore, this qualitative study will allow us to explore some potential intersectional findings related to gender, age or nationality, for example, to understand why such a low rate of refugees was included. Moving forward, new studies should evaluate simple and feasible PSG approaches and evaluate, with a feasible yet robust study design, how they work (or do not work) and what their impact is in humanitarian settings. Furthermore, it is important to include the voices of those living with diabetes and hypertension in future PSG programme and evaluation design. Specifically, new studies should prioritize mixed methods approaches with larger sample sizes, extended follow-up periods, and patient-centred outcomes that are relevant to humanitarian settings and are feasible to measure.

## Conclusion

We showed that it is feasible to implement PSGs in humanitarian settings for people living with hypertension or diabetes. While our findings indicate some improvement in quality-of-life following PSG implementation, we did not find statistical evidence of improvements on clinical outcomes in comparison with a control group. Further studies should evaluate the implementation of PSG in a way that is well accepted by local stakeholders, including humanitarian actors and people living with NCDs in these contexts. It is important to better understand which aspects of PSGs are effective (or not) in humanitarian settings and to design studies that are both robust, and adaptable to these challenging environments.

## Electronic supplementary material

Below is the link to the electronic supplementary material.

Supplementary Material 1

## Data Availability

The dataset used and/or analysed from the current study are available from corresponding author on reasonable request.
