# Supplementary material for: Evaluation of a peer support group programme for vulnerable host population and refugees living with diabetes and/or hypertension in Lebanon: a before-after study
Source: Confl Health. 2025 Jan 29;19:5. doi: 10.1186/s13031-025-00646-4 (PMC11776164; doi:10.1186/s13031-025-00646-4)
Supplement: Supplementary file 1 — Supplementary Material 1 [file 13031_2025_646_MOESM1_ESM.docx]

**ANNEX - SUPPLEMENTARY MATERIAL**

**Figure S1:** Mean SBP of control and PSG participants over time for both waves

**Table S1:** Crude and adjusted association between W1 PSG and change in SBP for Hypertensives

|  | **SBP** | |
| --- | --- | --- |
|  | **Beta (95%CI)** | **aBeta (95%CI)** |
| **Intervention** |  |  |
| Control | - | - |
| PSG | -4.59 [-12.34 to 3.15] | -0.16 [-7.27 to 6.94] |
|  |  |  |
| Baseline SBP (mmHg) | **-0.52 [-0.64 to -0.40]** | **-0.53 [-0.65 to -0.40]** |
|  |  |  |
| **Sex** |  |  |
| Male | - | - |
| Female | -0.59 [-4.90 to 3.72] | -2.36 [-6.31 to 1.60] |
|  |  |  |
| **Age/10 (years)** | -0.65 [-2.97 to 1.67] | -0.45 [-2.52 to 1.61] |

**Table S2:** Crude and adjusted association between W1 PSG and change in SBP and HbA1c for Diabetics

|  | **SBP** | | **HbA1c** | |
| --- | --- | --- | --- | --- |
|  | **Beta (95%CI)** | **aBeta (95%CI)** | **Beta (95%CI)** | **aBeta (95%CI)** |
| **Intervention** |  |  |  |  |
| Control | - | - | - | - |
| PSG | **-12.75 [-24.11 to -1.40]** | -8.14 [-21.75 to 5.48] | 0.43 [-0.48 to 1.35] | 0.11 [-0.82 to 1.05] |
|  |  |  |  |  |
| Baseline SBP (mmHg) | **-0.48 [-0.76 to -0.20]** | **-0.42 [-0.73 to -0.1]** |  |  |
|  |  |  |  |  |
| Baseline HbA1c (%) |  |  | **-0.34 [-0.53 to -0.14]** | **-0.32 [-0.51 to -0.12]** |
|  |  |  |  |  |
| **Sex** |  |  |  |  |
| Male | - | - | - | - |
| Female | -1.03 [-11.98 to 9.92] | 1.13 [-10.58 to 12.85] | 0.78 [-0.03 to 1.59] | 0.46 [-0.41 to 1.34] |
|  |  |  |  |  |
| **Age/10 (years)** | 0.01 [-7.57 to 7.59] | -0.90 [-8.00 to 6.20] | 0.17 [-0.41 to 0.75] | 0.14 [-0.39 to 0.67] |

**Table S3:** Crude and adjusted association between W1 PSG and change in SBP and HbA1c for patients with both Diabetes and Hypertension

|  | **SBP** | | **HbA1c** | |
| --- | --- | --- | --- | --- |
|  | **Beta (95%CI)** | **aBeta (95%CI)** | **Beta (95%CI)** | **aBeta (95%CI)** |
| **Intervention** |  |  |  |  |
| Control | - | - | - | - |
| PSG | 1.10 [-9.17 to 11.37] | 0.23 [-8.39 to 8.86] | -0.39 [-0.81 to 0.04] | **-0.47 [-0.90 to -0.03]** |
|  |  |  |  |  |
| Baseline SBP (mmHg) | **-0.74 [-0.95 to -0.53]** | **-0.74 [-0.96 to -0.53]** |  |  |
|  |  |  |  |  |
| Baseline HbA1c (%) |  |  | -0.11 [-0.25 to 0.01] | **-0.15 [-0.28 to -0.02]** |
|  |  |  |  |  |
| **Sex** |  |  |  |  |
| Male | - | - | - | - |
| Female | 3.54 [-6.85 to 13.94] | -0.19 [-9.06 to 8.68] | **0.06 [-0.38 to 0.50]** | 0.31 [-0.13 to 0.75] |
|  |  |  |  |  |
| **Age/10 (years)** | -1.22 [-7.06 to 4.61] | 0.32 [-4.4 to 5.05] | 0.21 [-0.03 to 0.45] | **0.36 [0.11 to 0.60]** |

**Table S4:** Crude and adjusted association between W2 PSG and change in SBP for Hypertensives

|  | **SBP** | |
| --- | --- | --- |
|  | **Beta (95%CI)** | **aBeta (95%CI)** |
| **Intervention** |  |  |
| Control | - | - |
| PSG | 1.8 [-6.82 to 10.41] | 3.27 [-3.99 to 10.53] |
|  |  |  |
| Baseline SBP (mmHg) | **-0.56 [-0.67 to -0.44]** | **-0.57 [-0.69 to -0.45]** |
|  |  |  |
|  |  |  |
| **Sex** |  |  |
| Male | - | - |
| Female | -1.06 [-6.04 to 3.92] | 1.80 [-2.38 to 5.98] |
|  |  |  |
| **Age/10 (years)** | -1.06 [-3.59 to 1.46] | 0.82 [-1.35 to 2.98] |

**Table S5:** Crude and adjusted association between W2 PSG and change in SBP and HbA1c for Diabetics

|  | **SBP** | | **HbA1c** | |
| --- | --- | --- | --- | --- |
|  | **Beta (95%CI)** | **aBeta (95%CI)** | **Beta (95%CI)** | **aBeta (95%CI)** |
| **Intervention** |  |  |  |  |
| Control | - | - | - | - |
| PSG | 3.91 [-4.47 to 12.29] | 5.14 [-2.78 to 13.07] | 0.12 [-0.69 to 0.92] | 0.06 [-0.67 to 0.80] |
|  |  |  |  |  |
| Baseline SBP (mmHg) | **-0.29 [-0.45 to -0.13]** | **-0.31 [-0.48 to -0.14]** |  |  |
|  |  |  |  |  |
| Baseline HbA1c (%) |  |  | **-0.26 [-0.41 to -0.11]** | **-0.26 [-0.42 to -0.10]** |
|  |  |  |  |  |
| **Sex** |  |  |  |  |
| Male | - | - | - | - |
| Female | 1.07 [-5.56 to 7.71] | -1.69 [-8.03 to 4.66] | 0.23 [-0.39 to 0.87] | -0.14 [-0.75 to 0.46] |
|  |  |  |  |  |
| **Age/10 (years)** | 0.68 [-2.62 to 3.98] | 0.39 [-2.70 to 3.49] | 0.42 [0.12 to 0.72] | **0.38 [0.09 to 0.67]** |

**Table S6:** Crude and adjusted association between W2 PSG and change in SBP and HbA1c for patients with both Diabetes and Hypertension

|  | **SBP** | | **HbA1c** | |
| --- | --- | --- | --- | --- |
|  | **Beta (95%CI)** | **aBeta (95%CI)** | **Beta (95%CI)** | **aBeta (95%CI)** |
| **Intervention** |  |  |  |  |
| Control | - | - | - | - |
| PSG | -2.12 [-10.02 to 5.77] | -0.08 [-7.03 to 6.86] | 0.23 [-0.31 to 0.78] | 0.26 [-0.18 to 0.71] |
|  |  |  |  |  |
| Baseline SBP (mmHg) | **-0.44 [-0.59 to -0.29]** | **-0.48 [-0.63 to -0.32]** |  |  |
|  |  |  |  |  |
| Baseline HbA1c (%) |  |  | **-0.47 [-0.59 to -0.34]** | **-0.46 [-0.59 to -0.34]** |
|  |  |  |  |  |
| **Sex** |  |  |  |  |
| Male | - | - | - | - |
| Female | -2.68 [-9.96 to 4.59] | **-7.39 [-14.00 to -0.77]** | -0.28 [-0.78 to 0.22] | -0.13 [-0.55 to 0.29] |
|  |  |  |  |  |
| **Age/10 (years)** | 0.26 [-3.68 to 4.21] | -0.15 [-3.65 to 3.34] | -0.16 [-0.43 to 0.11] | -0.15 [-0.37 to 0.08] |
